# Identification and characterization of *CONSTANS*-like (*COL*) gene family in upland cotton (*Gossypium hirsutum* L.)

**DOI:** 10.1371/journal.pone.0179038

**Published:** 2017-06-07

**Authors:** Darun Cai, Hui Liu, Na Sang, Xianzhong Huang

**Affiliations:** Special Plant Genomics Laboratory, College of Life Sciences, Shihezi University, Shihezi, Xinjiang, China; National Taiwan University, TAIWAN

## Abstract

The *CONSTANS/FLOWERING LOCUS T* (*CO/FT*) regulon plays a central role in the control of flowering time in photoperiod-sensitive plants. Flowering time in wild cotton (*Gossypium* spp.) has strict photoperiod sensitivity, but domesticated cotton is day-neutral. Information on the molecular characterization of the *CO* and *CO*-like (*COL*) genes in cotton is very limited. In this study, we identified 42 *COL* homologs (*GhCOL*s) in the *G*. *hirsutum* genome, and many of them were previously unreported. We studied their chromosome distribution, phylogenetic relationships, and structures of genes and proteins. Our results showed that GhCOLs were classified into three groups, and 14 COLs in group I showed conserved structure when compared with other plants. Two homoeologous pairs, *GhCOL1-A* and *GhCOL1-D* in Group I, showed the highest sequence similarity to *Arabidopsis thaliana CO* and rice *CO* homologous gene *Heading date1* (*Hd1*). Tissue-specific expression showed that 42 *GhCOL* genes may function as tissue-specific regulators in different cells or organs. We cloned and sequenced the 14 *GhCOL* genes in Group I related to flowering induction to study their diurnal expression pattern, and found that their expression showed distinct circadian regulation. Most of them peaked at dawn and decreased rapidly to their minima at dusk, then started to accumulate until following dawn under long- or short-day conditions. Transgenic study in the *Arabidopsis co-2* mutant demonstrated that *GhCOL1-A* and *GhCOL1-D* fully rescued the late-flowering phenotype, whereas *GhCOL3-A*, *GhCOL3-D*, *GhCOL7-A*, and *GhCOL7-D* partially rescued the late-flowering phenotype, and the other five homoeologous pairs in Group I did not promote flowering. These results indicate that *GhCOL1-A* and *GhCOL1-D* were potential flowering inducers, and are candidate genes for research in flowering regulation in cotton.

## Introduction

Seasonal and diurnal variations of day length in nature are consistent from year to year. Many plants perceive photoperiodic information to predict upcoming environmental changes and precisely regulate flowering time in favorable conditions [1]. In plants, the circadian clock regulates a wide range of biological processes and represents the plant’s endogenous timekeeper. Two proteins, CONSTANS (CO) and FLOWERING LOCUS T (FT), are the central integrator of the photoperiod pathway in *Arabidopsis thaliana* [2]. *AtCO* induces the expression of *FT* in the leaf under long-day (LD) inductive conditions [1,3,4]. In rice, *heading date 1* (*Hd1*, the *CO* ortholog) promotes *heading date 3a* (*Hd3a*, the *FT* ortholog) expression under short-day (SD) conditions, but inhibits *Hd3a* expression under non-inductive LD conditions [5]. Many studies have shown that flowering time is governed by the CO/FT module which is highly conserved among photoperiod-sensitive plants although its action models are inconsistent in different species [6–8]. *CO* encodes a putative B-box zinc finger transcription factor unique to plants and mediates between the circadian clock and the flowering time control [9–11]. High CO levels activate the expression of *FT*, which encodes a member of the phosphatidylethanolamine-binding protein that is a major component of florigen [3,4,12].

It has been documented that the accumulation of *CO* mRNA and CO protein is regulated at the transcriptional and posttranslational level through a number of proteins. Cycling of *CO* mRNA is regulated transcriptionally through circadian clock-regulated components, such as GIGANTIA (GI), CYCLING DOF FACTORS (CDFs), and the F-box protein FLAVIN BINDING, KELCHREPEAT (FKF1) [13–17]. The GI-FKF1 complex modulates CO protein stability, which degrades a family of CO repressors, the CDFs, resulting in maximum *CO* transcription at the end of the day [11,18]. Plants can perceive specific light quality by multiple photoreceptors to trigger posttranslational regulation of CO protein. In the early morning under LD conditions, the red-light receptor phytochrome B (PHYB) promotes degradation of CO protein and plays a major role in the regulation early in the day [19,20]. The E3 ubiquitin ligase HIGH EXPRESSION OF OSMOTICALLY RESPONSIVE GENES1 (HOS1) that physically interacts with CO is involved in the red light-mediated degradation of CO that occurs early in the daylight period [21,22]. In the evening, blue light prevents CO proteolysis by CONSTITUTIVE PHOTOMORPHOGENIC1 (COP1) [19,23,24]. The far-red receptor phytochrome (PHYA) and the blue-light receptors Cryptochrome 1 (CRY1) and CRY2 stabilize CO protein toward the end of the day through inhibition of proteasome-dependent CO degradation [25,26,27].

CONSTANS-like (COL) proteins in this family are characterized by the presence of one or two zinc finger B-box domains at the N-terminus or a C-terminal CCT (CO, CO-like, and TOC1) domain [10]. The *COL* gene family in both monocots and dicots has many members, for example 17 in *Arabidopsis* [28], 16 in rice [29], 9 in barley [30], 10 in sugar beet [31], 11 in *Medicago* [32], 26 in soybean [33], 25 in Chinese cabbage [34], 11 in *Chrysanthemum lavandulifolium* [35], 6 in ramie [36], and 25 in banana [37]. Phylogenetic analysis divided COL proteins in plants into three major groups [30]. Group I COLs contain two B-box domains, one CCT domain, and an additional VP motif (valine-proline motif involved in the interaction with COP1). Group II COLs contain only one B-box and a CCT domain. Group III have one full B-box, a second diverged zinc finger, and a CCT domain [30,38,39].

The cotton genus (*Gossypium*) contains approximately 50 species and five allopolyploid species [40]. Wild cotton species are perennial plants and mostly SD-photoperiodic, with a diversity of architecture and flowering time. However, domesticated cotton species underwent extensive artificial selection and gradually lost their photoperiodic sensitivity. Upland cotton (*G*. *hirsutum* L.) is the most extensively cultivated *Gossypium* species and numerous elite types have been bred successfully, which have been widely grown in more than 80 countries and account for more than 95% of commercial cotton production worldwide [41,42]. However, the molecular mechanisms regulating the transition from vegetative to reproductive growth in cotton are less well characterized than in other plant species, mostly due to the complexity of the cotton genome and scarcity of cotton flowering time mutants. Zhang et al. [43] reported identification of 23 putative *COL* genes in *G*. *raimondii* based on its genome sequence data. They studied their structures, phylogenetic relationships, and molecular evolution, and found that *COL1*, *COL2*, and *COL8* experienced greater selective pressures during the domestication process [43]. To date, information on the numbers and characterizations of *COL* genes in *G*. *hirsutum* is not clear. However, successful sequencing of the *G*. *hirsutum* genome provides a valuable resource for genome evolution, fiber improvement, and gene identification [44,45]. Because of the lack of good information on the numbers and characterizations of *COL* genes in *G*. *hirsutum*, we aim to characterize *COL* family members in *G*. *hirsutum* using its genome sequence data. We identified and characterized 42 *GhCOL* genes and their chromosomal distribution, phylogenetic relationship, gene structure, conserved motif, and tissue specificity expression profiles. Additionally, we focused on the 14 *GhCOLs* in Group I–which has been characterized in many plant species–and this cluster with *AtCO* and rice *Hd1*. We respectively examined the diurnal expression of the 14 *GhCOLs* under LD or SD conditions. We further performed complement experiments to analyze their putative functions in the flowering signal pathway. Our results support the conclusion that *GhCOL1-A* and *GhCOL1-D* homoeologs may be the key inducers of flowering in cotton. Our data also provide a broader understanding of the *COL* gene family in upland cotton.

## Materials and methods

### Plant material and growth conditions

Cotton seeds (*G*. *hirsutum* L. cv. XLZ 42) were field-grown under natural conditions during the summer of 2015 in Shihezi (Xinjiang, China). The seeds of *Arabidopsis* ecotype L*er and* mutant *co-2* (in the L*er* background) obtained from the Arabidopsis Biology Resources Center (ABRC, Columbus, OH, USA) were surface sterilized for 20 min with 2.8% sodium hypochlorite solution containing 0.1% surfactant (Triton X-100; Sigma-Aldrich, Munich, Germany) and rinsed several times with sterile water. The sterilized seeds were stratified for 3 d at 4°C in darkness and then plated on Petri dishes with half-strength Murashige-Skoog (MS) salt mixture (pH 5.7; Duchefa, Haarlem, the Netherlands), 1% (w/v) sucrose, and 0.8% (w/v) agar. Petri dishes were then placed in a phytotron at 22°C for 10 d under LD conditions (16 h light/8 h dark), and the seedlings were transplanted into pots containing peat soil and vermiculite (1:1) and kept in a growth chamber with a 16-h photoperiod. The light intensity for *Arabidopsis* growth was 200 μmol m^–2^ s^–1^.

For tissue expression analysis, roots, stems, leaves, and shoot apical meristems (SAM) were collected at the third true-leaf expanding stage (approximately 20 d after planting). During the cotton flowering period, tissues of sepals and petals were collected at 0 d of anthesis (DOA), and fibers were sampled at 15 d post-anthesis (DPA). For diurnal rhythmic expression analyses, the plants were grown in a 25°C chamber in LD and SD conditions (8 h light/16 h dark photoperiod) with 150 μmol m^–2^ s^–1^ of light intensity, respectively. The third true leaves were sampled every 4 h at 13 different time points from zeitgeber time (ZT) 0 h for 2 d. For gene expression analyses, the fresh leaves of 20 d L*er*, *co-2* and all the transgenic lines were sampled under LD conditions. All samples were frozen immediately in liquid nitrogen and stored at –80°C.

### Identification of *COL* family genes from *G*. *hirsutum*

In an effort to identify all *COL* family genes in the upland cotton genome, a batch Basic Local Alignment Search Tool (BLAST) search was performed against the *G*. *hirsutum* genome (v1.1) [45] downloaded from CottonGen (https://www.cottongen.org/) using the full-length amino acid sequences of *Arabidopsis* CO and *G*. *raimondii* COLs [43] as queries with an *E*-value cut of 1 × 10^−15^. All retrieved proteins were then submitted to PFAM (http://pfam.xfam.org/) databases for annotating of the domain structure. Only candidates encoding both one or two zinc-binding B-box domains at the N-terminus and a CCT domain at the C terminus were regarded as “true” *G*. *hirsutum* COs (GhCOLs). The Blast search continued until no more new COL homologs were matched. As a result, 42 genomic sequences of *GhCOL*s were obtained. We found that *GhCOL1-A* and *GhCOL8-A* genes were not annotated in the *G*. *hirsutum* genome (v1.1) [45], whereas their homoeologous *GhCOL1-D* and *GhCOL8-D* genes were. Therefore, we amplified the coding sequences of *GhCOL1-A* and *GhCOL8-A* by PCR using gene-specific primers based on the *GhCOL1-D* and *GhCOL8-D* sequences. The detailed information of the upland cotton *COL* genes was supplied in S1 Table. We found that no sequences of *GhCOL2-A*, *GhCOL2-D*, *GhCOL18-D*, and *GhCOL23-D* were annotated in the *G*. *hirsutum* genome database, and so these four *GhCOL* genes were not identified.

### Chromosomal mapping and phylogenetic analysis

Chromosomal position and gene structure information of *GhCOLs* were obtained from *G*. *hirsutum* gene annotation (v1.1) [45], and these putative *COL* genes were mapped on the corresponding A_t_ (‘t’ indicates tetraploid) or D_t_ chromosomes using the MapInspect software (http://mapinspect.software.informer.com/). In total, 122 *COL* homologs (S2 Table), including 16 *Arabidopsis COL*s, 14 rice *COL*s, 26 soybean *COL*s, 23 *G*. *raimondii COL*s, and 42 *GhCOL*s were used to construct a phylogenetic tree. Multiple sequence alignments were performed by ClustalW [46] under default parameters with a gap opening penalty of 10 and gap extension penalty of 0.2. MEGA5.1 [47] was used to make a phylogeny reconstruction analysis using the Neighbor-Joining (NJ) method and Poisson correction distance model. The bootstrap analysis was performed to estimate nodal support on the basis of 1000 re-samplings.

### Gene structure and protein profile analysis

Gene exon–intron structure information for *GhCOL*s was retrieved from *G*. *hirsutum* gene annotation (v1.1) [45], and a gene structure schematic diagram was drawn using the Gene Structure Display Server [48]. Protein length, molecular weight, and isoelectric point of GhCOLs were analyzed using Lasergene v7.1 software (http://www.dnastar.com/) with default parameters. Protein subcellular localization was predicted by WoLF PSORT (www.genscript.com/wolf-psort.html).

### RNA preparation, cDNA synthesis, and qRT-PCR analyses

Total RNA was isolated using the RNAprep pure Plant Kit (Tiangen, Beijing, China) according to the manufacturer’s protocol. The quality and quantity of each RNA sample were determined using gel electrophoresis and a NanoDrop 2000 spectrophotometer (NanoDrop Technologies, Wilmington, DE, USA). The cDNA synthesis reactions were performed using the Superscript First-Strand Synthesis System (Invitrogen, Carlsbad, CA, USA) according to the manufacturer’s instructions with 1 μg of total RNA per reaction used as a template.

Quantitative real-time PCR (qRT-PCR) was carried out on an Applied Biosystems 7500 Fast Real-Time PCR System (Life Technologies, Foster City, CA, USA) in a 25-μl volume containing 10 ng of cDNA, 5 pM of each primer, and 25 μl of Fast SYBR Green Master Mixture (CWBIO, Beijing, China) according to the manufacturer’s protocol. The PCR conditions were as follows: primary denaturation at 95°C for 20 s followed by 40 amplification cycles of 3 s at 95°C, and 30 s at 60°C. Melting curve analysis was performed to ensure there was no primer-dimer formation. Amplicons were also loaded on a 2% agarose gel for visual inspection. Primer information of qRT-PCR for gene expression analysis and gene cloning used in this study is listed in S3 Table. The nucleotide sequences of *GhCOLs* in Group I marked with primer location for qRT-PCR were shown in S1 Fig. Three replicate assays were performed with independently isolated RNAs, and each RT reaction was loaded in triplicate. Relative expression levels of each *GhCOL* gene are presented using the 2^–Δ*C*t^ method [49].

The heatmap of tissue expression for all *GhCOLs* was performed as described by Deng et al. [50]. All the 2^–Δ*C*t^ values calculated from *C*t data by qRT-PCR in different tissues, including root, stem, true leaf, flower, sepal, SAM, and fiber were saved in a Microsoft Excel spreadsheet (.xls). This file can be loaded into a Heat map Illustrator tool named HemI 1.0 (http://hemi.biocuckoo.org/) for visualizing a heatmap of gene expression. Given a selected color scale, the total color space will be automatically processed into a numerical matrix. HemI project contains all information needed to draw a heatmap and will generate a heatmap after loaded. Last, a publication-quality heatmap of gene expression can be exported directly.

### Cloning of *GhCOL* genes in Group I and transformation of *Arabidopsis*

The complete open reading frame cDNAs for seven pairs of homoeologs in Group I were obtained from *G*. *hirsutum* cv. XLZ42 by PCR amplification using gene-specific primers designed according to the putative A- or D-homoeologous sequences in *G*. *hirsutum* genome database (S3 Table), and then subcloned into the pMD-19 vector (TaKaRa, Dalian, China) following the manufacturer’s instructions. Several independent clones for each *COL* gene were sequenced for validation of A- or D-homoeologous *COL*s by comparing sequences with TM-1 genome. Finally, 14 coding sequences of *COL*s were separately transferred into the overexpression binary vector *pCAMBIA 2300-35S-OCS* [51] to construct *35S*:*GhCOL*s. The later flowering *Arabidopsis co-2* mutant plants were separately infected with *Agrobacterium tumefaciens* strain GV3101 transformed with the obtained *35S*:*GhCOL*s clones using the floral dip method [52]. Transgenic plants were selected on half-strength MS culture medium containing 50 μg/ml kanamycin. Homozygotes were replanted and subsequently monitored for flowering using non-transgenic wild type seedlings as controls. Flowering time was recorded as the number of rosette leaves per plant at the time the first flower bloomed from at least 20 individuals for each T_3_ lines and control [53]. Statistical analysis of the number of rosette leaves was performed using Student’s *t*-test.

## Results

### Identification and chromosomal distribution of *COL* family genes in upland cotton

To identity the *COL* family genes in the upland cotton genome, we carried out a genome-wide analysis of the putative *GhCOL* genes in the TM-1 genome database. We obtained 42 putative genomic sequences of upland *COL* homologs, and each *GhCOL* was then assigned a name based on its similarity level to *Arabidopsis CO* and *COL*s (S1 Table), with a designation of A or D for A- or D-subgenome chromosome. The methods of classification and nomenclature for *G*. *hirsutum GhCOL*s in our study were consistent with *G*. *raimondii COL*s [43].

Subcellular localization prediction showed that most GhCOL proteins mainly located in nucleus or both nucleus and cytoplasm, which are correlated to their functions as transcription factors (S1 Table). However, GhCOL3, GhCOL5 and GhCOL6 homoeologous protein located only in cytoplasm, and GhCOL17 homoeologs located only in chloroplast.

**Fig 1:**
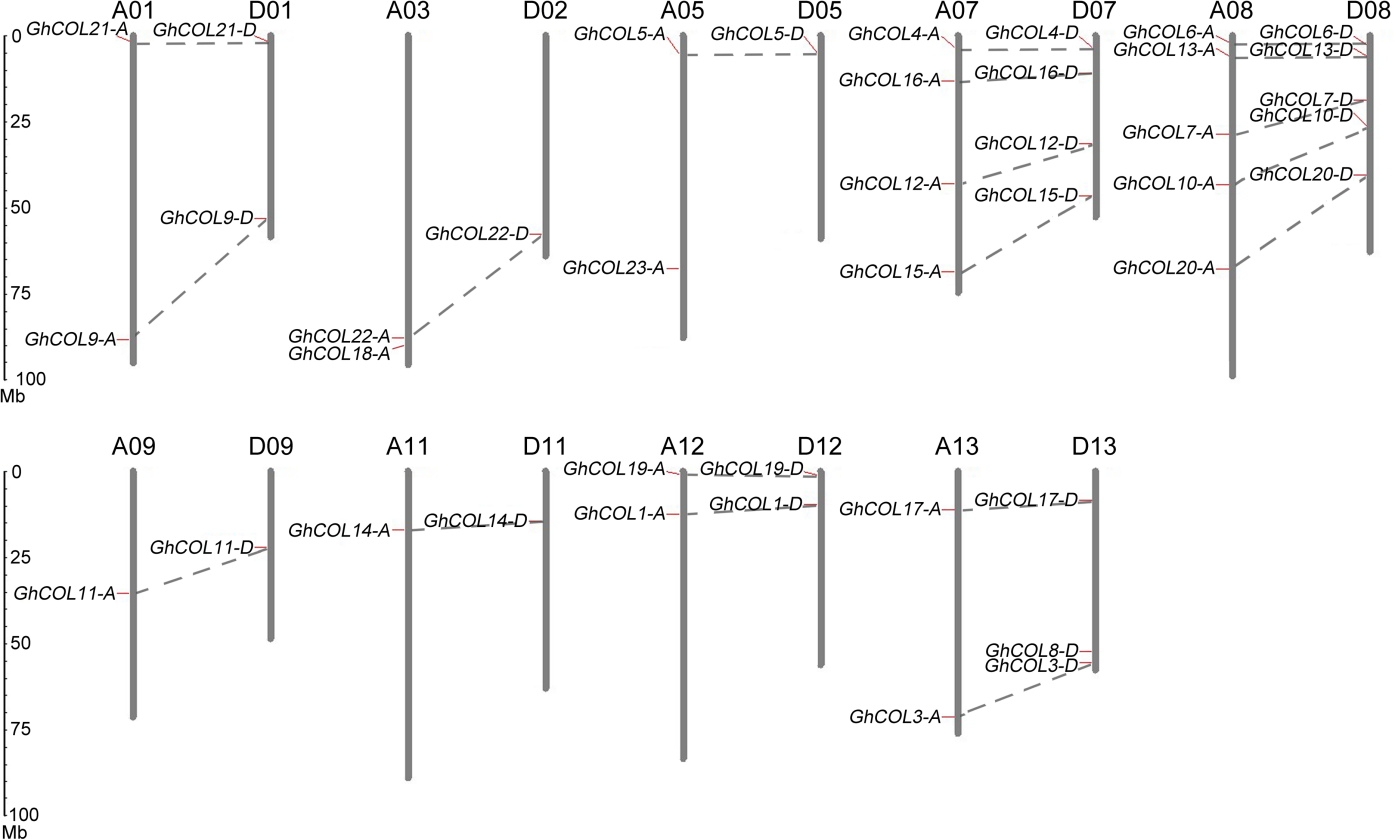
Chromosomal distributions of the identified *CONSTANS-*like (*COL*) genes in upland cotton (*G*. *hirsutum* acc. TM-1). Chromosomal locations were shown from top to bottom on corresponding chromosomes according to *G*. *hirsutum* genome (v1.1) annotation [45]. Duplicated gene pairs were linked by dotted lines.

Chromosome mapping reveals that the 42 *GhCOL*s were not evenly distributed on the 18 chromosomes (Fig 1). There were 1–5 genes on each chromosome: one gene on chromosomes D02, D05, A09, D09, A11, and D11; two genes on chromosomes A01, D01, A03, A05, A12, D12, and A13; three genes on chromosome D13; four genes on chromosomes A 07 and D 07; and five genes on chromosomes A 08 and D 08. The distribution ratio for each chromosome was in the range of 2.38–11.91%.

### Phylogenetic tree, gene structure, and conserved motif analyses of *GhCOL*s

**Fig 2:**
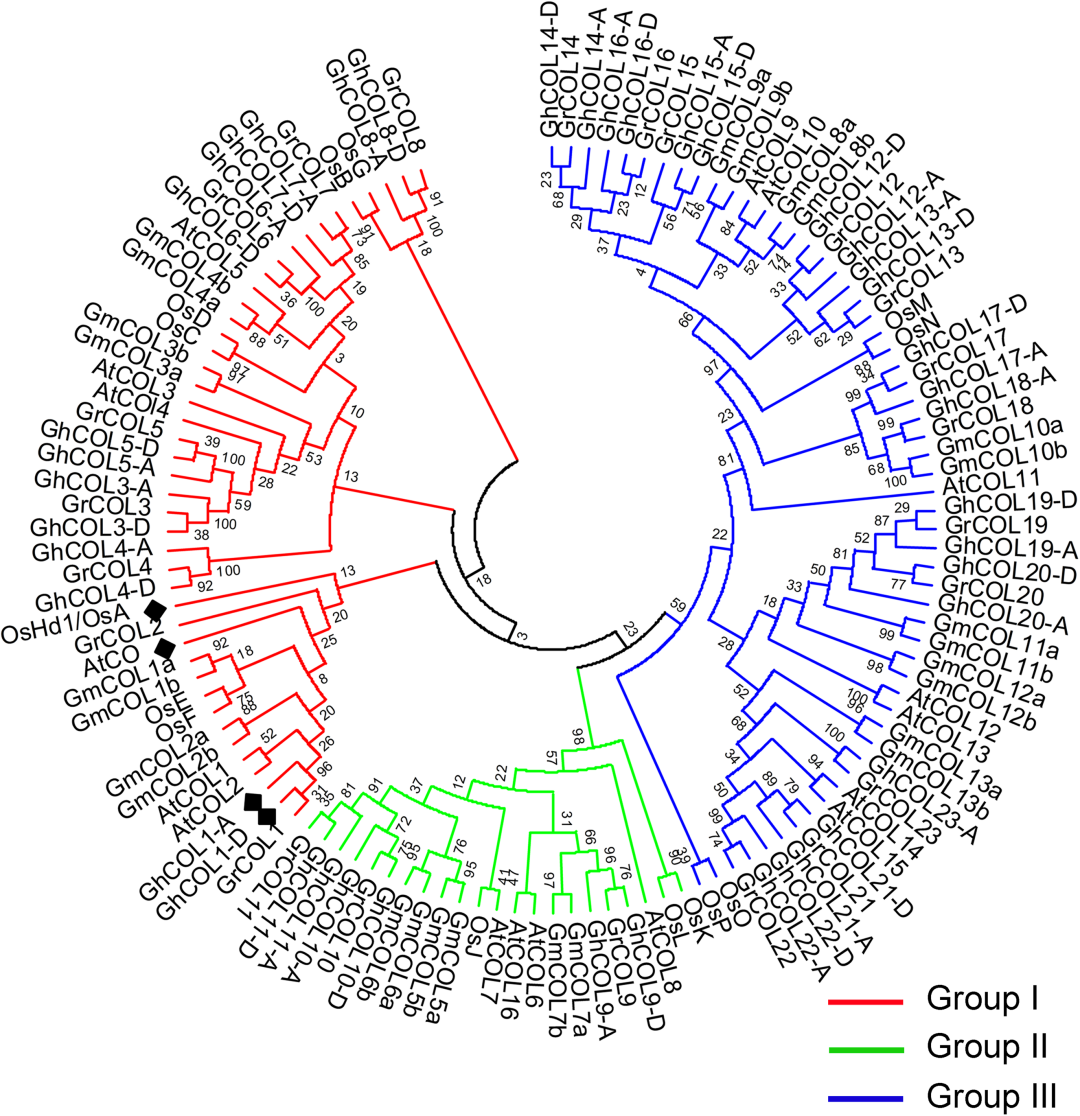
**A Neighbor Joining phylogenetic analysis of the CO and COLs family from *Arabidopsis* (At), rice (Os), soybean (Gm), *G*. *raimondii* (Gr) and *G*. *hirsutum* (Gh).** Multiple alignments were generated using ClustalW [46]. The phylogenetic tree was constructed using MEGA5.1 [47]. Bootstrap values for 1,000 re-samplings were shown on each branch. The 122 CO homologs from five plant species were identified by homology searches in GenBank database using *Arabidopsis* CO and COL proteins as entry. The clades were divided into three groups, branches of which were marked in differently colors. AtCO, OsHd1, GhCOL1-A and GhCOL1-D were indicated using a black prism.

To investigate the phylogenetic relationships among *COL* family genes, we constructed a NJ phylogenetic tree with 122 COL protein amino-acid sequences retrieved from *Arabidopsis*, rice, soybean, and cotton databases based on multiple alignment analyses. These *COL* homologs were classified into three major clades, and cotton *COL*s were divided into Group I–III (Fig 2), consistent with results for diploid cotton (*G*. *raimondii*) [43]. Of the 42 *GhCOL*s in *G*. *hirsutum*, 14 genes were in Group I, six were in Group II, and the remaining 22 were in Group III. Among the 14 GhCOLs in Group I, the homoeologs of GhCOL1-A and GhCOL1-D had 98.7% amino acid sequence similarity and were clustered with the known functional flowering inducers, *Arabidopsis* CO [10] and rice Hd1 [29].

**Fig 3:**
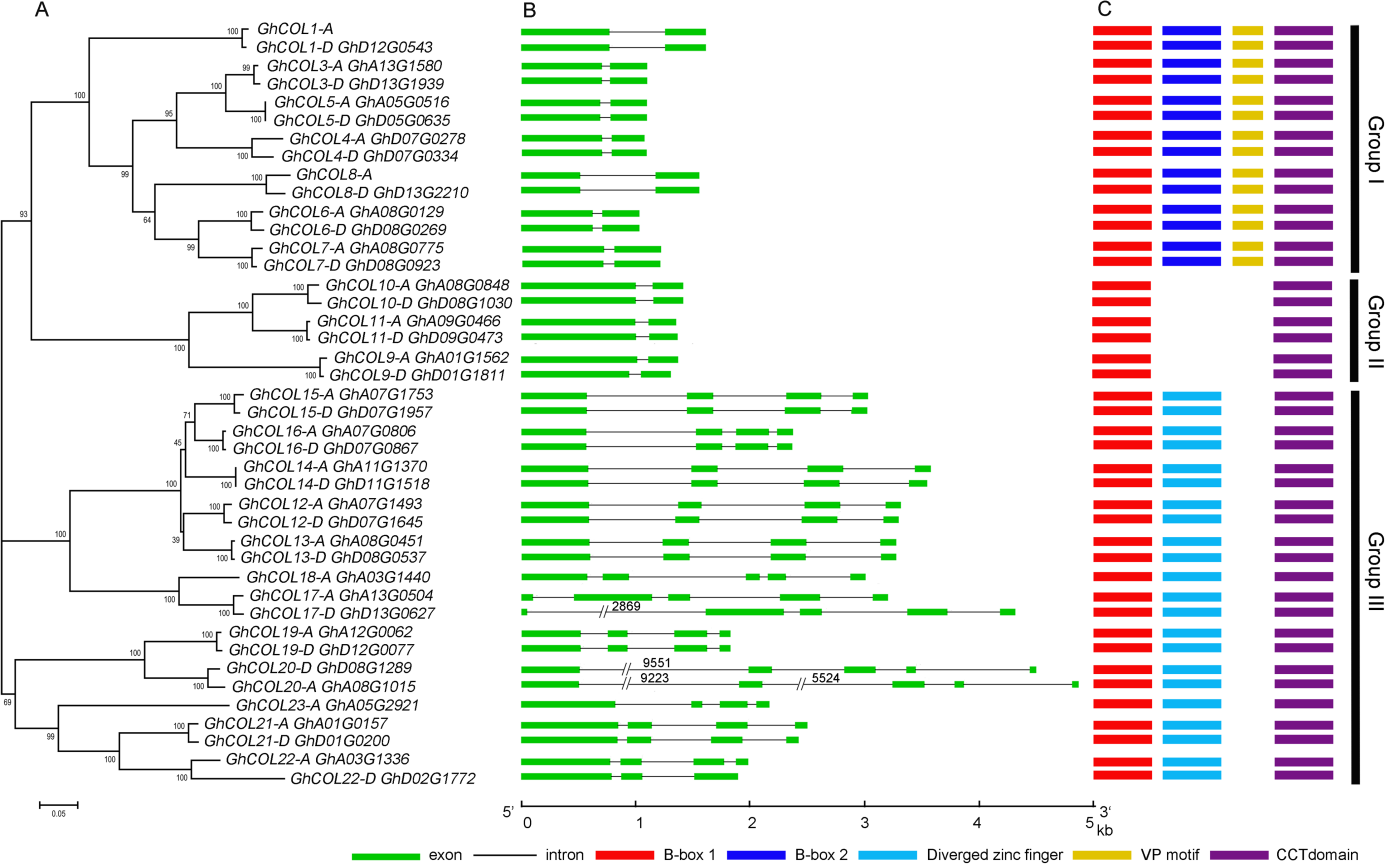
Phylogenetic relationships and structures of *GhCOL* genes and GhCOL proteins. **(A)** A Neighbor-Joining (NJ) phylogenetic tree of the 42 *COL* homologs from *G*. *hirsutum* was constructed using MEGA5.1 [47]. The bootstrap consensus tree was inferred from 1,000 replicates. (B) The gene structures were drawn using the Gene Structure Display Server [48]. Green boxes and black lines were exonic and intronic regions, respectively. (C) The domain structure of GhCOL proteins. Colorful boxes indicated B-box 1, B-box 2, diverged zinc finger, VP motif and CCT domain, respectively.

Phylogenetic analysis of 42 GhCOL proteins showed that cotton GhCOLs were categorized into three groups more obviously (Fig 3A). We analyzed the genome structure of the 42 *GhCOL* genes by aligning the genomic and cDNA sequences (Fig 3B). The 14 genes in Group I and six in Group II were highly conserved, containing two exons and one intron, and their full-length genomic DNA sequences ranged from 1,031 bp (*GhCOL6-D*) to 1,611 bp (*GhCOL1-D*). Of the 14 *COL*s in Group I, the intron lengths in *GhCOL1-D* and *GhCOL8-D* were obviously longer than in other family members, whereas exon I in *COL8-D* was shorter than others, leading to variation in gene length. However, 17 genes in Group III had different gene structure. *GhCOL17-A*, *GhCOL17-D*, *GhCOL18-A*, *GhCOL20-A*, and *GhCOL20-D* contained five exons, while the other 12 genes contained four exons. The genome lengths of these 17 genes ranged from 1,824 bp (*GhCOL19-A* and *GhCOL19-A*) to 5,613 bp (*GhCOL17-D*) except for *GhCOL20-A* and *GhCOL20-D* which had an abnormally-sized first intron.

The annotation of the domain structure and multiple alignments of amino acid sequences showed that the COLs of Group I contained one B-box 1, one B-box 2, one VP motif, and one CCT domain. However, B-box 1 in GhCOL6-A and GhCOL6-D, and B-box 1 and the VP motif in GhCOL8-A and GhCOL8-D, were incomplete. Six COLs in Group II had one B-box 1 and one CCT domain, whereas the remaining 22 COLs in Group III contained one B-box 1, one diverged zinc finger, and one CCT domain (Fig 3C and S2 Fig).

### Tissue-specific expression patterns of *GhCOL*s in upland cotton

**Fig 4:**
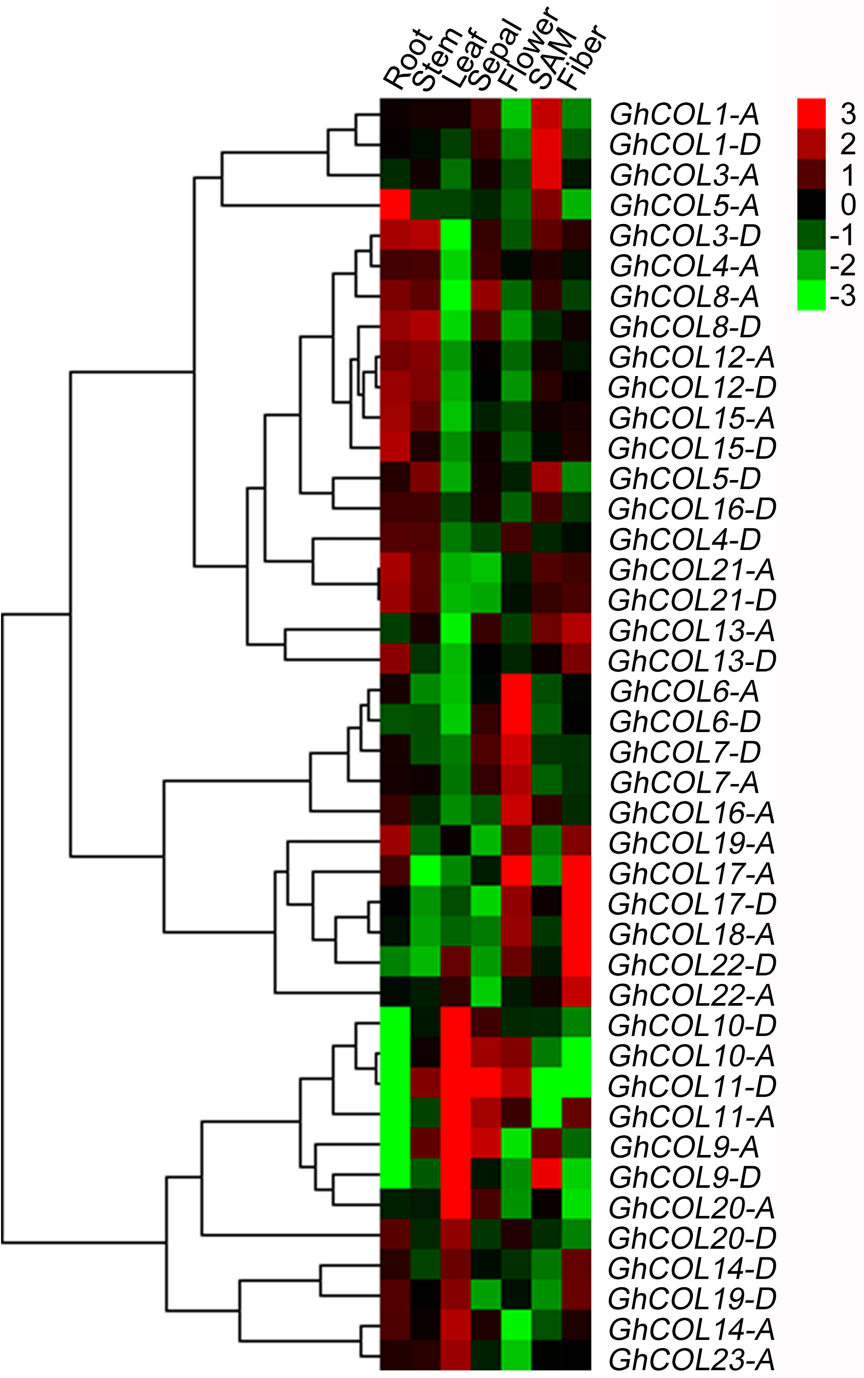
Heat map of *GhCOL* genes expression profiles in cotton different tissues. Quantitative real time-PCR (qRT-PCR) was used to analyze the relative expression levels of 42 *GhCOL* genes in various tissues, and cotton *UBQ7* (GenBank accession no. DQ116441) was used as an internal control. Roots, stems, leaves and shoot apical meristems (SAM) were sampled at the third true-leaf stage, and sepals were collected at the flowering stage, respectively. Fibers were sampled on 15 d post anthesis (DPA). The patterns were clustered and visualized using heatmap program HemI 1.0 [50]. The color scale at the right-above of the heat map is given in log^2^ -transformed 2^–Δ*C*t^ value.

To understand the temporal and spatial transcriptional patterns of *GhCOL*s, we first analyzed their transcriptional levels in different tissues, including root, stem, true leaf, flower, sepal, SAM, and fiber using qRT-PCR. There were 42 *GhCOL*s expressed in various tissues with different expression levels (Fig 4). The expression patterns of *GhCOL*s were not consistent with their phylogenetic relationship of clustering into Group I–III (Fig 2). *GhCOL5-A*, *GhCOL12-D*, *GhCOL15-A*/*D*, and *GhCOL21-A*/*D* were mainly expressed in roots. *GhCOL3-D*, *GhCOL5-D*, *GhCOL8-D*, and *GhCOL12-A*/*D* were mainly expressed in stems. *GhCOL9-A*/*D*, *GhCOL10-A*/*D*, *GhCOL11-A*/*D*, and *GhCOL20-A* were predominantly expressed in leaves; and *GhCOL14-A/D*, *GhCOL19-D*, *GhCOL20-D*, and *GhCOL23-A* were also expressed in leaves with low expression. *GhCOL9-A* and *GhCOL11-D* were expressed significantly in sepals, and *GhCOL9-D* was also highly expressed in SAM. The *GhCOL6* and *GhCOL7* homoeologs, *GhCOL16-A*, and *GhCOL17-A*, were highly expressed in flowers, and *GhCOL17-A* was also highly expressed in fibers. The highest expressions of *GhCOL1* homoeologs and *GhCOL3-A* were only in SAM. *GhCOL17* homoeologs, *GhCOL18-A* and *GhCOL22-D*, were highly expressed in fibers. *GhCOL4* homoeologs and *GhCOL16-D* showed very low expression in various tissues. Our results showed that *GhCOL*s had specific transcript accumulation in seven different tissues, suggesting that they may function as tissue-specific regulators in different cells or organs of cotton.

### Diurnal expression pattern of Group I *GhCOLs* in LD and SD conditions

**Fig 5:**
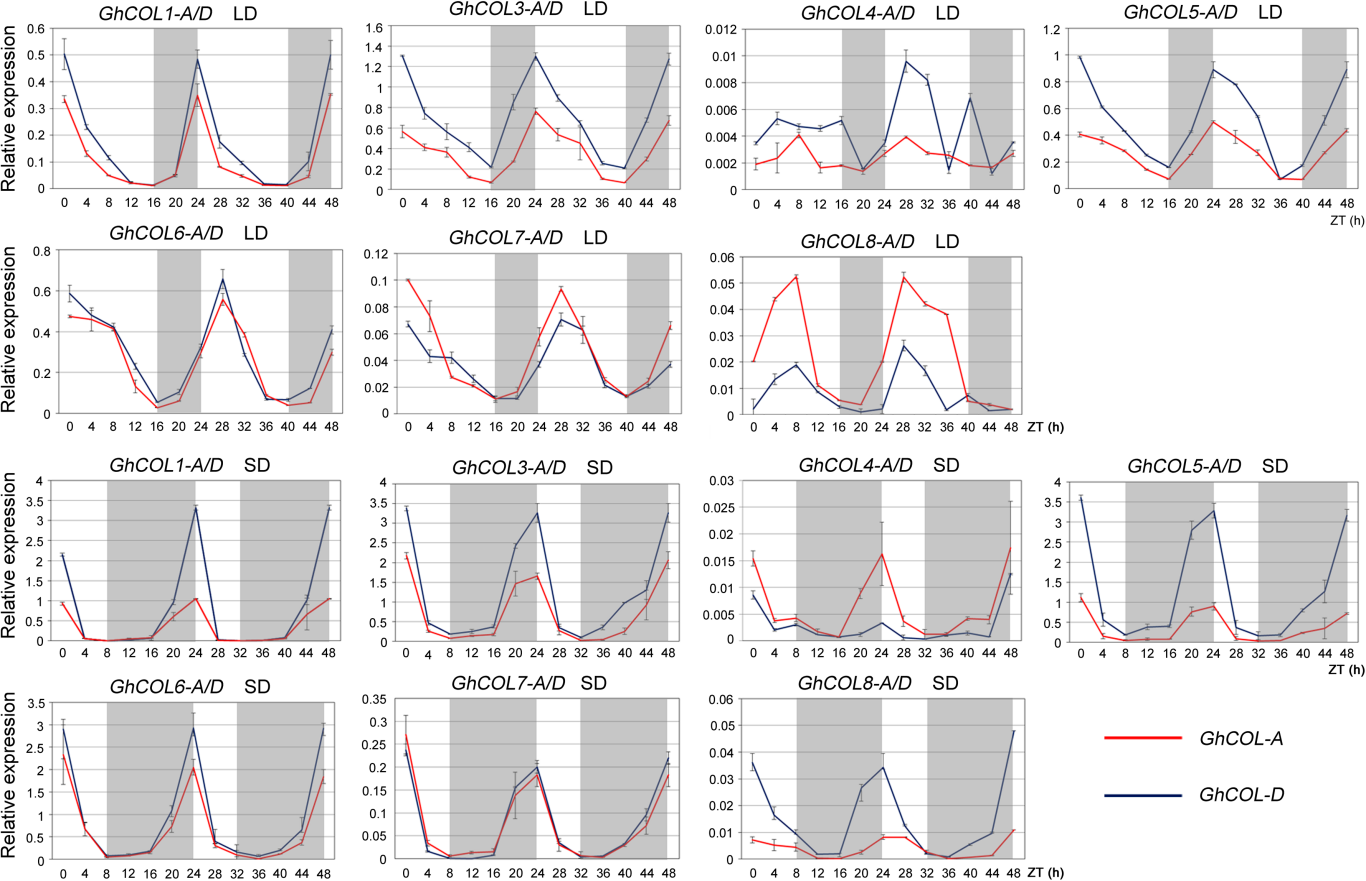
Diurnal expression pattern of the seven homoeologous *COL* gene pairs form Group I under LD or SD conditions. Sample collection started at the beginning of the light period at zeitgeber time (ZT) 0 and continued every 4 h for 48 h in LD and SD conditions. The x-axis shows the time points and y-axis represents relative gene expression against cotton *UBQ7* (DQ116441) as the control. Gray boxes over each chart indicate night. Data represent the mean ± SE obtained from three independent biological repeats.

*COL* genes in Group I have been documented to play key roles in regulating flowering time and show obvious circadian rhythm characteristics in all plants studied [37]. We next focused on investigating the seven pairs of homoeologous *COL* genes in Group I for respective diurnal expression patterns over a 48-h period at 4-h intervals in LD or SD conditions. In both light conditions, six homoeologous gene pairs, except for *GhCOL4-A*/*D* in LD, showed a clear diurnal expression pattern with biased A- or D-homoeologs expression (Fig 5). *GhCOL1*, *GhCOL3*, and *GhCOL5* homoeologs exhibited similar diurnal rhythms, and their expression peaked at dawn and then decreased rapidly to a minimum at dusk, then began to increase until the following dawn.

Under LD, *GhCOL6* and *GhCOL7* homoeologs showed cyclic expression patterns with light/dark induction treatment, but the expression peak occurred at dawn or 4 h later. Interestingly, under SD both showed similar expression patterns with peaks at dawn and a rapid decline to their minima, which was similar to *Arabidopsis CO* under SD conditions [11]. The *COL4* and *COL8* homoeologs showed obviously different expression patterns in both light conditions. In LD, *COL4-D* expression peaked more than twice, and *COL4-A* peaked once. However, in SD, *COL4* homoeologs had clear diurnal expression similar to *COL1*, *COL3* and *COL5* homoeologs. Expression of *COL8* homoeologs peaked at dawn under SD but 4 h later for LD.

### Ectopic expression of *GhCOL1-A* and *GhCOL1-D* promotes flowering in *Arabidopsis*

**Fig 6:**
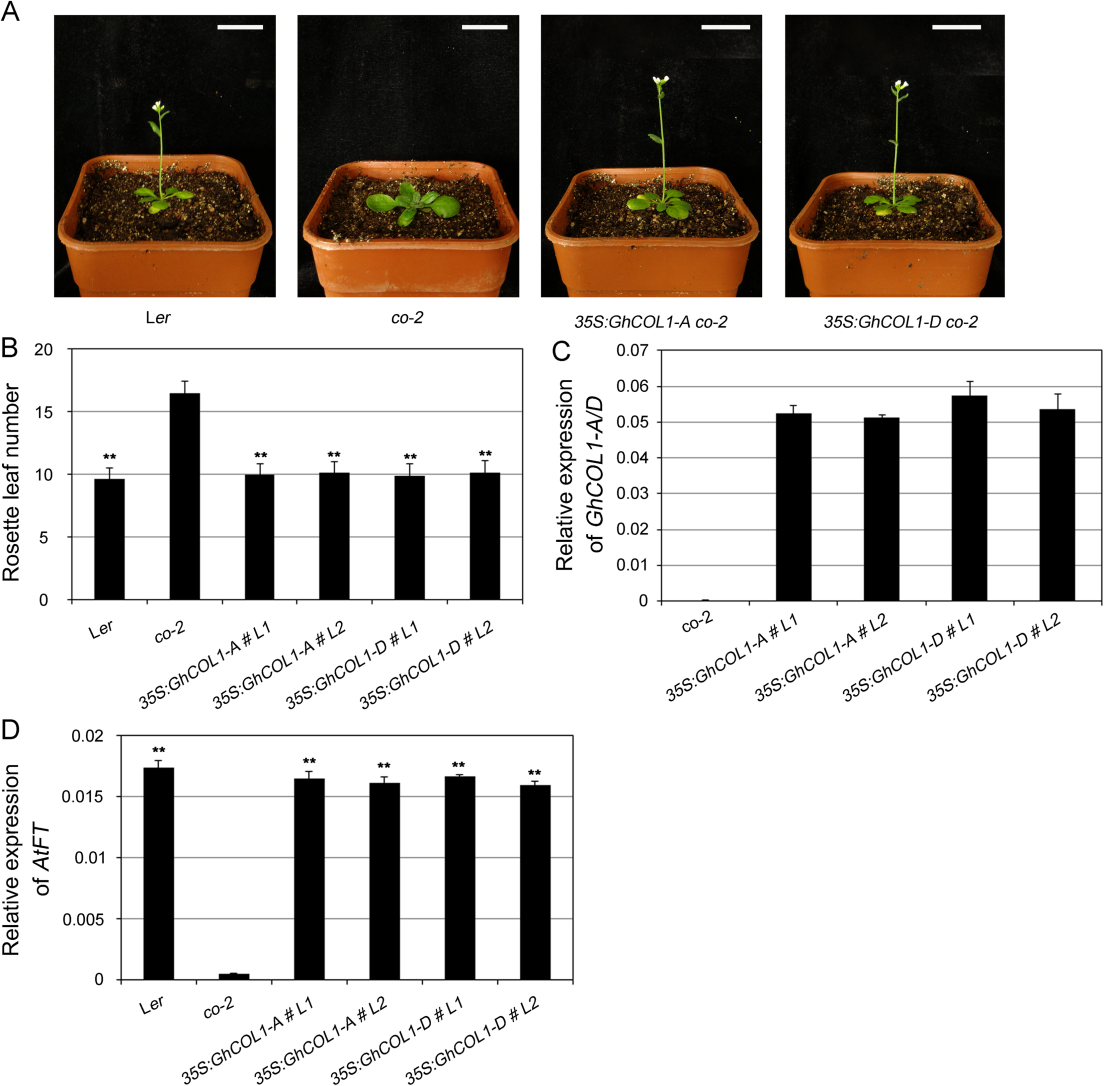
Overexpression of *GhCOL1-A* and *GhCOL1-D* rescued the late-flowering phenotype of the *Arabidopsis co-2* mutant. (A) Representative phenotype of 20 d L*er*, *co-2*, *35S*:*GhCOL1-A* and *35S*:*GhCOL1-D* transgenic line in phytotron under LD conditions. Scale bar, 1 cm. (B) Flowering time was measured as the rosette leaves number per plant. Data represent a minimum of 10 plants for each line ± SE. (C) Detection of *GhCOL1-A/D* expression by qRT-PCR in *35S*:*GhCOL1s* transgenic lines and *co-2* under LD conditions. (D) The expression level of endogenous *AtFT* (*AT1G65480*) was determined by qRT-PCR. Data represent the mean ± SE (n = 3) obtained from three independent biological repeats in (C) and (D), and *AtACT 2* (*AT3G18780*) was used as internal control. ** indicate significant differences compared with *co-2* at *P* < 0.01 according to the Student’s *t*-test.

To further explore possible roles in flowering control of cotton homoeologous genes derived from past whole-genome duplication events, we cloned the coding sequences of the seven homoeologous *COL* gene pairs in Group I from *G*. *hirsutum* cv. XLZ42. Multiple alignments of amino acid sequences among 14 cotton GhCOLs and *Arabidopsis* AtCO and rice Hd1 are shown in S3 Fig. We then expressed them under the CaMV *35S* promoter in *co-2* mutant *Arabidopsis*. While *co-2* mutant *Arabidopsis* exhibited obvious late flowering compared to wild-type (L*er*) under LD, the transgenic plants expressing *GhCOL1* homoeologs flowered significantly earlier than *co-2* mutants (Fig 6A), with similar rosette leaf numbers to L*er* (Fig 6B), and *GhCOL* genes were confirmed to be overexpressed in the transgenic *co-2* plants by qRT-PCR (Fig 6C and Figure C in S4 Fig). In the *co-2* mutant, expression of endogenous *AtFT* was hardly detectable compared with basal levels in L*er*. However, *AtFT* transcripts in transgenic plants approached the level detected in the L*er* background (Fig 6D). These results showed that ectopic expression of *GhCOL1* homoeologs complemented the late-flowering effect of *co-2*. We also found that the flowering times in transgenic plants expressing *GhCOL3* and *GhCOL7* homoeologs were also slightly earlier than *co-2*, but still later than wild-type under the same conditions (Figures A and B in S4 Fig). The endogenous *AtFT* transcripts exceeded levels in the *co-2* mutant, but were far below the levels in L*er* (Figure D in S4 Fig), suggesting that *GhCOL3* and *GhCOL7* homoeologs partially complemented the later flowering phenotype of *co-2*. However, overexpression of *GhCOL4*, *GhCOL5*, *GhCOL6*, and *GhCOL8* homoeologous gene pairs had no influence on flowering time of *co-2* (S4 Fig).

## Discussion

### Functional conservation and divergence of *COL* gene family in cotton

Many studies have shown that the CO/FT regulon in photoperiod-responsive plant species plays an important role in regulating flowering transition, but our understanding of the molecular mechanism is still limited, especially in polyploid species. Upland cotton is a domesticated allotetraploid and is cultivated worldwide, and has gradually lost their photoperiod sensitivity. The CO/FT module in cultivated cotton remains unclear. In total, 42 *GhCOL*s family genes from the *G*. *hirsutum* genome (acc. TM-1) were identified and characterized in the present study. They were distributed unevenly along 18 different chromosomes (Fig 1), and phylogenetic analysis clustered them into three groups (Fig 2 and Fig 3A). Both gene structures and the conserved protein motifs of GhCOLs shared high similarity with known COL homologs involved in photoperiod-responsive plant species, suggesting that the function of *COL* family genes was highly conserved during evolution of a wide range of plant species (Fig 3B and 3C).

Fourteen COL proteins in Group I in upland cotton had two B-box, one VP motif, and one CCT domain (Fig 3C and S2 Fig). Zhang et al [43] analyzed the expression levels of eight *COL* genes derived from TM-1 in Group I, and found that they all had diurnal expression patterns. In Zhang’s study, however, qRT-PCR primers used for gene expression detection did not discriminate between A- or D-subgenomes, and so the expression patterns of homoeologous genes were not clear. Due to polyploidization, expression levels of many homoeologous genes are unequal in allotetraploid cotton [45]. To understand their possible roles, we performed a detailed transcript-level characterization of seven homoeologous *COL* genes pairs in Group I. We analyzed the diurnal expression patterns of the A- and D-homoeologs in detail by designing gene-specific primers based on their single nucleotide polymorphism, showing a clear expression of diurnal rhythm for all 14 genes in cv. XLZ 42, consistent with published data [43].

*COL1*, *COL3*, and *COL5* homoeologs showed similar diurnal expression patterns under both light conditions, with more consistent expression rhythm in SD (Fig 5B), and their expression peaked at dawn and declined rapidly to minima at dusk. Under LD, *COL6*, *COL7*, and *COL8* homoeologs had clear cyclic expression patterns, and their expression peaks occurred at dawn 4 h later; whereas under SD, their expression patterns were similar to *COL1*, *COL3*, and *COL5* homoeologs. Under LD, the peak times for *COL4* homoeologs differed from each other; whereas the expression rhythms in SD were also similar to *COL1*, *COL3*, and *COL5* homoeologs. Among seven *COL* homoeologs in upland cotton, there were slightly more genes with expression bias toward D_t_ than toward A_t_ homoeologs, consistent with published data [45]. In summary, the diurnal expression analyses indicated that in photoperiodic flowering, cotton *COL* family genes in Group I had similar or conserved functions. Unequal expression of *COL* homoeologs between A_t_ and D_t_ subgenomic loci may lead to subfunctionalization or neofunctionalization in allotetraploid cotton, but detailed functional analyses of cotton *COL* family genes are still needed.

In addition to regulating flower times, the *COL* gene family is involved in a wide range of events in plant development in response to photoperiodic signaling, including seedling growth [54,55], dormancy [7], tuberization [56], and cell growth [38]. Functional divergence of the *COL* gene family in *Arabidopsis* has been frequently reported. For example, *AtCO*, *AtCOL1*, and *AtCOL2* share high sequence similarity. However, altered expression of *AtCOL1* and *AtCOL2* in transgenic plants accelerated the circadian clock, but had little effect on flowering time [57]. Unlike *AtCO*, *AtCOL3* represses flowering and influences root growth and lateral root formation [54]. The expression of *AtCOL9* is also regulated by the circadian clock in the photoperiod pathway. Unexpectedly, *AtCOL9* overexpression repressed flowering through repression of *AtCO* as well as *AtFT* [58]. Diverse diurnal expression patterns of the *GhCOL* family genes strongly suggested functional divergence of cotton *COL* homoeologs in multiple aspects of photoperiodic response, including flowering.

Furthermore, tissue-specific expression patterns also strongly indicated that multiple functions of *GhCOL*s were not necessarily related to flowering. Although 42 *COL* genes were expressed in all examined tissues, the average expression levels and numbers of expressed genes varied among the seven different tissues. *GhCOL1* homoeologs and *GhCOL3-A* were solely highly expressed in the SAM. *GhCOL5-A* was predominantly expressed in roots. *GhCOL9-D*, *GhCOL10* homoeologs, and *GhCOL20-A* were solely highly expressed in leaves. *GhCOL6*, *GhCOL7* homoeologs, and *GhCOL16-A* were solely highly expressed in flowers. These data suggest specific functions in root, leaf, flower, and SAM for specific *COL* genes, whereas the similar expression patterns suggest functional redundancy, and biased-expressed homoeologous *COLs* genes may lead to diverse functionalization. In addition, *GhCOL17* homoeologs, *GhCOL18-A* and *GhCOL22-D*, were highly expressed in fibers, suggesting involvement in fiber development. Their functional divergence and exact roles in cotton growth require further study.

### GhCOL1-A and ChCOL1-D are potential flowering inducers and activators of *GhFT1* in *G*. *hirsutum*

Of the 42 cotton COLs, we explored which COL homoeologs were the flowering inducers in cotton. We gathered evidence indicating that the GhCOL1-A and GhCOL1-D homoeologs were the flowering inducers in *G*. *hirsutum*. First, GhCOL1-A was shown to have 55.1 and 43.5% amino acid sequence similarity with the *Arabidopsis* CO and rice Hd1, which both function as flowering inducers, while correspondingly, GhCOL1-D had 55.6 and 45.3% similarity (S3 Fig). Second, phylogenetic analysis indicated that *GhCOL1-A* and *GhCOL1-D* clustered together with *AtCO* and *Hd1* (Fig 2). Third, *GhCOL1-A* and *GhCOL1-D* mRNA abundance showed similar oscillations under both LD and SD conditions, and the highest levels of mRNA were at dawn (Fig 5), showing similarity with *Arabidopsis CO* [11]. The *GhCOL1-A* and *GhCOL1-D* mRNA levels continued to oscillate for a period of 24 h, indicating that they were regulated by the circadian clock. Last, our transgenic study showed that overexpression of *GhCOL1-A* and *GhCOL1-D* rescued the late-flowering phenotype of the *Arabidopsis* loss-of-function *co-2* mutant, thereby demonstrating their crucial role in flowering (Fig 6). Moreover, by over-expressing *GhCOL1-A*, or *GhCOL1-D*, endogenous *AtFT* transcription was almost fully restored to the normal levels (Fig 6D).

Our previous study showed that under LD or SD conditions, the expression pattern of *GhFT1* (*FT* ortholog of *G*. *hirsutum*) was rhythmic with an expression peak 4 h into the light period [52]. The 4-h time lag between the expression peak of *GhCOL1* homoeologs and *GhFT1* suggest a putative novel mechanism in cotton CO/FT regulation.

Taken together, the results show that *GhCOL1-A* and *GhCOL1-D* were the potential activator of *GhFT1*, and the CO/FT module reported in *Arabidopsis*, rice, and other plants was conserved in *G*. *hirsutum*. We suggest that *GhCOL1* homoeologs play important roles in flowering regulation of cotton in response to changing photoperiod. Further experiments will clarify the molecular mechanism and explore the functions of other *GhCOL* homoeologs.

## Supporting information

S1 Fig Primers location of *GhCOLs* in Group I for qRT-PCR. Left and right black arrows indicated the locations of forward and reverse primers, respectively. Red frames indicated the differences of nucleotides between *GhCOL-A* and *GhCOL-D* homoeologs in Group I.
(TIF)

S2 Fig Partial amino acid sequence alignment and conserved motifs of GhCOL proteins. Multiple alignments of amino acid sequences of 42 GhCOLs were performed using ClustalW [46]. (A) 14 COLs in Group I. (B) six COLs in Group II. (C) 22 COLs in group III. Conserved amino acids were highlighted in black and the similar in grey. The B-box1, B-box2, VP motif, zinc finger and CCT conserved sequences were marked with horizontal lines.
(TIF)

S3 Fig Multiple alignments of amino acid sequences of *Arabidopsis* AtCO, rice Hd1 and cotton GhCOL1s in Group I. AtCO (NP_1978088.1) and Hd1 (BAB17627.1) were retrieved from GenBank. Conserved amino acids are highlighted in black and the similar in grey. The gaps indicated by dashes are attributed to the lack of amino acids.
(TIF)

S4 Fig Overexpression of other *GhCOL*s in Group I influenced the late flowering phenotype of the *Arabidopsis co-2* mutant. (A) Representative phenotype of 20 days L*er*, *co-2* and transgenic plants grown in phytotron under LD conditions. Scale bar, 1 cm. (B) Flowering time was measured as the rosette leaves number per plant. Data represent a minimum of 10 plants scored for each line ± SE. (C) Detection of *GhCOLs* expression by qRT-PCR in *35S*:*GhCOLs* transgenic lines and *co-2* under LD conditions. (D) The expression level of *Arabidopsis FT* was determined by qRT-PCR. Data represent the mean ± SE from three biological replicates in (C) and (D), and *AtACT2* (*AT3G18780*) was used as internal control. ** and * indicate significant differences in comparison with *co-2* mutant at *P* < 0.01 and *P* < 0.05 according to the Student’s *t*-test compared to mutant, respectively.
(TIF)

S1 Table Profiles of *GhCOL* gene family in upland cotton. (XLSX)

S2 Table The COL homologs used as data set in phylogenetic analysis. (XLSX)

S3 Table Sequences of the primers used in this study. (XLSX)
