# Supplementary figures and images for: Identification and characterization of CONSTANS-like (COL) gene family in upland cotton (Gossypium hirsutum L.)
Source: PLoS One. 2017 Jun 7;12(6):e0179038. doi: 10.1371/journal.pone.0179038 (PMC5462432; doi:10.1371/journal.pone.0179038)

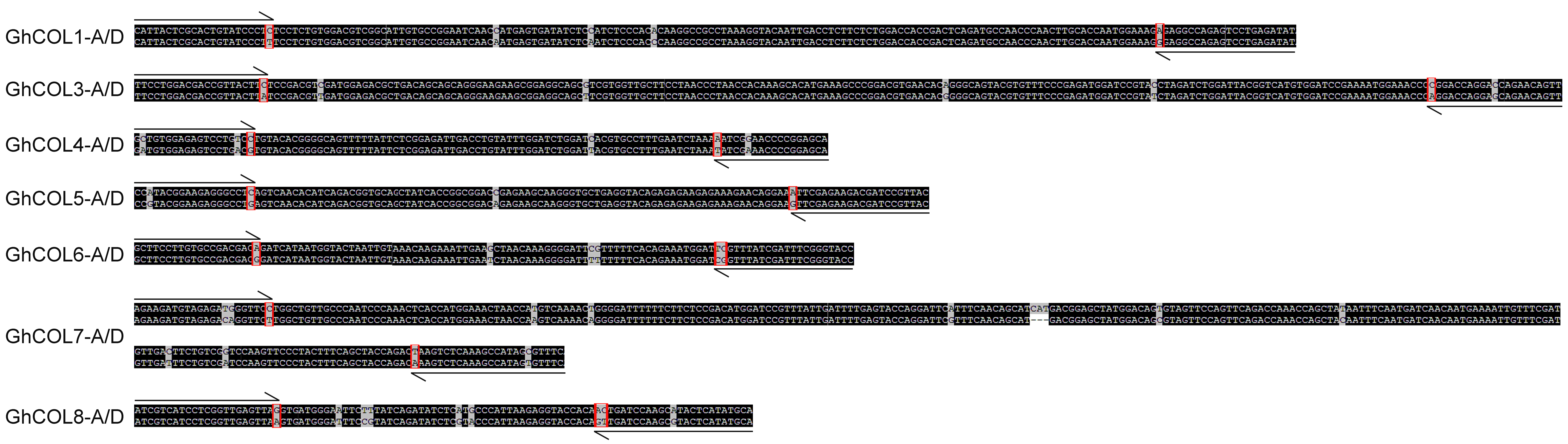

Supplement: S1 Fig — Left and right black arrows indicated the locations of forward and reverse primers, respectively. Red frames indicated the differences of nucleotides between GhCOL-A and GhCOL-D homoeologs in Group I. (TIF) [file pone.0179038.s001.tif]

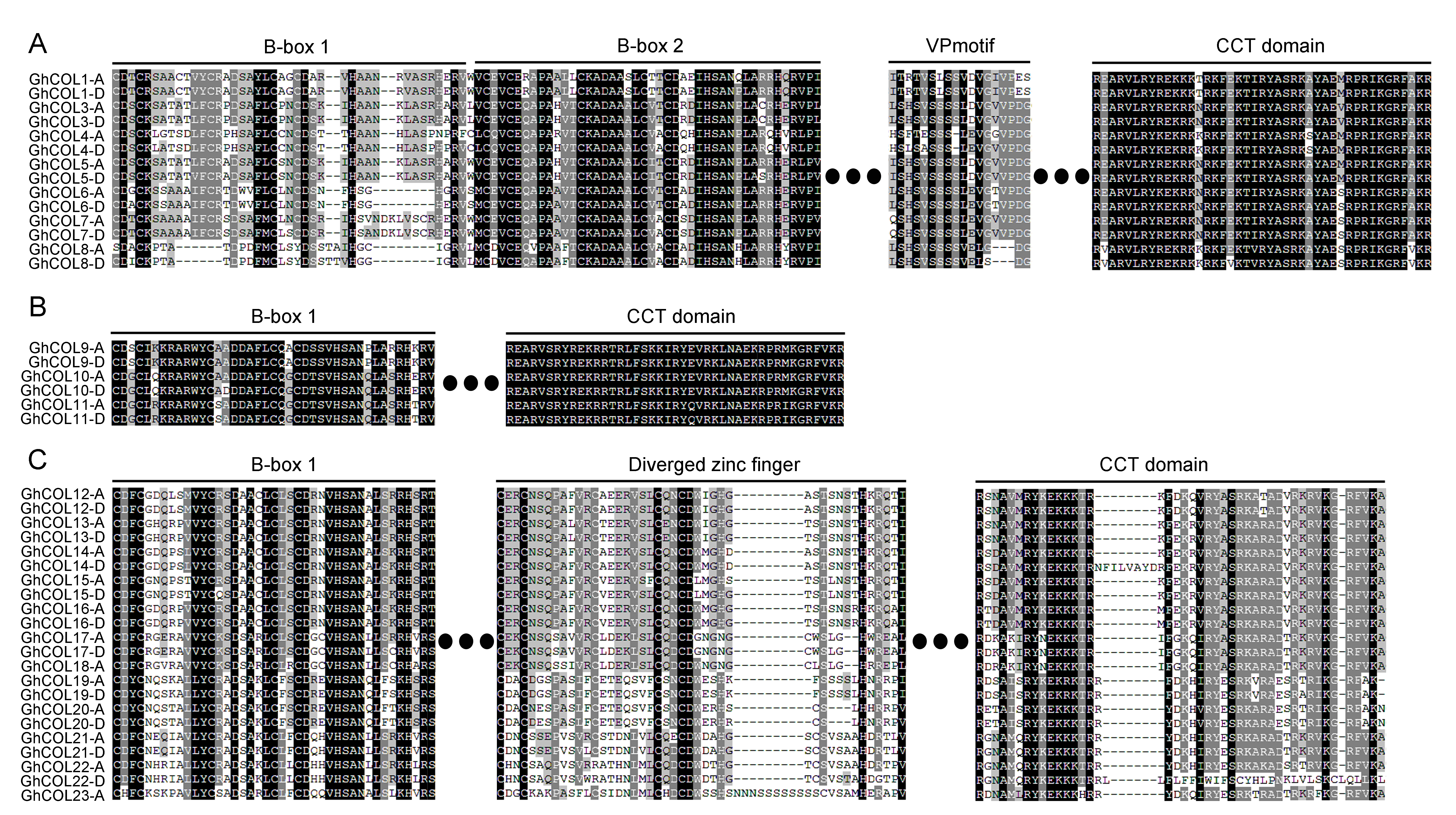

Supplement: S2 Fig — Multiple alignments of amino acid sequences of 42 GhCOLs were performed using ClustalW [46]. (A) 14 COLs in Group I. (B) six COLs in Group II. (C) 22 COLs in group III. Conserved amino acids were highlighted in black and the similar in grey. The B-box1, B-box2, VP motif, zinc finger and CCT conserved sequences were marked with horizontal lines. (TIF) [file pone.0179038.s002.tif]

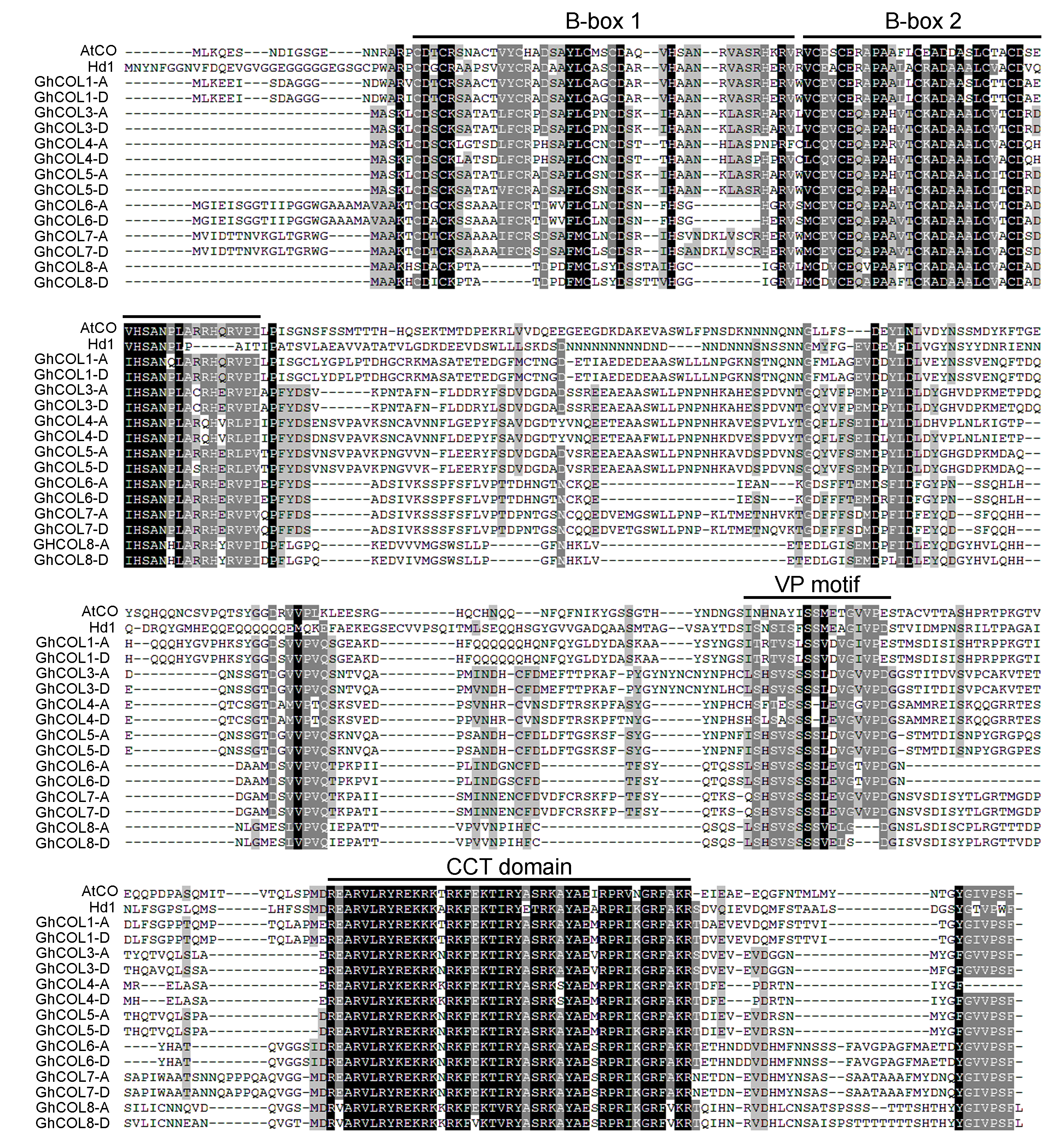

Supplement: S3 Fig — AtCO (NP_1978088.1) and Hd1 (BAB17627.1) were retrieved from GenBank. Conserved amino acids are highlighted in black and the similar in grey. The gaps indicated by dashes are attributed to the lack of amino acids. (TIF) [file pone.0179038.s003.tif]

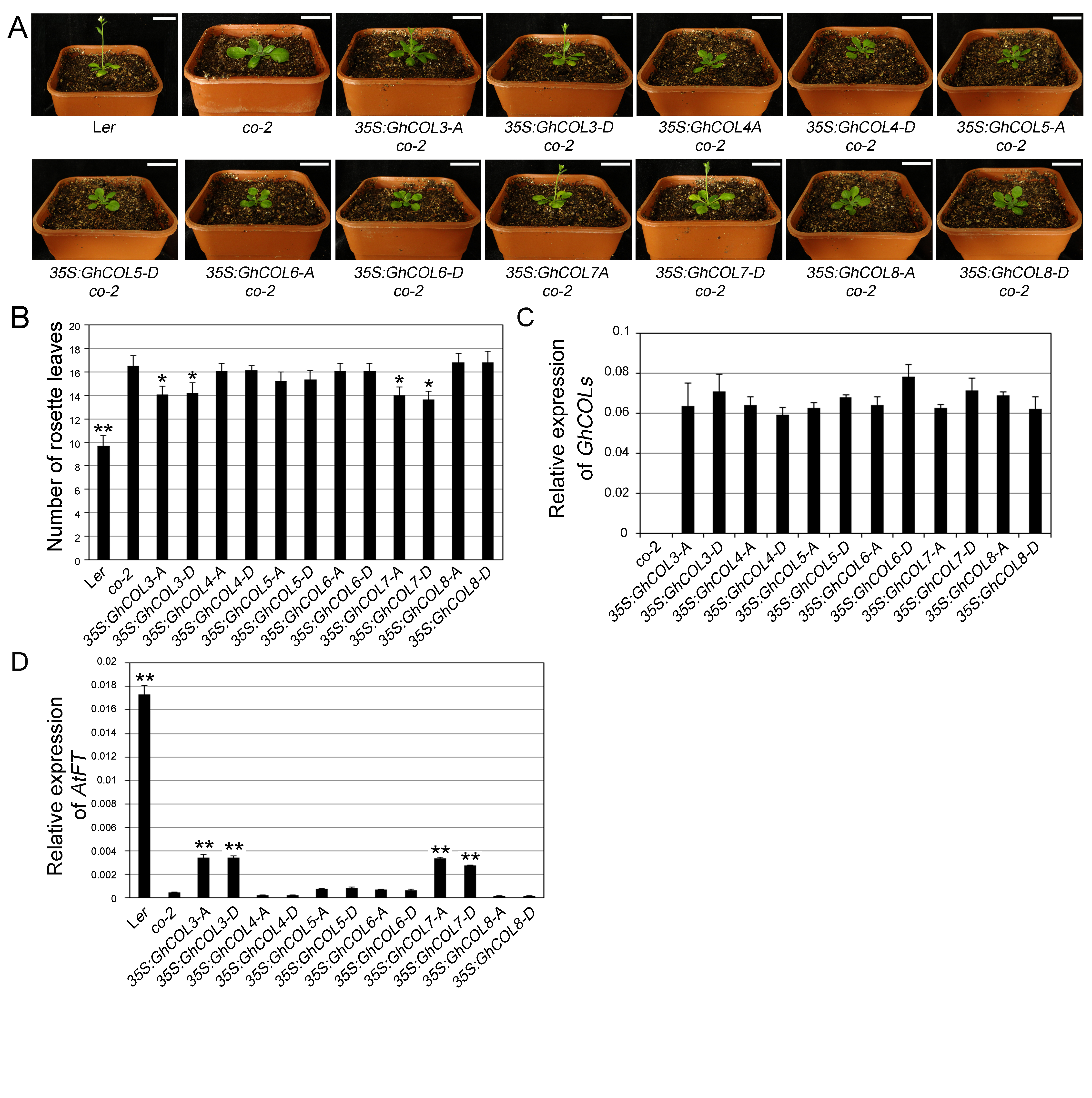

Supplement: S4 Fig — (A) Representative phenotype of 20 days Ler, co-2 and transgenic plants grown in phytotron under LD conditions. Scale bar, 1 cm. (B) Flowering time was measured as the rosette leaves number per plant. Data represent a minimum of 10 plants scored for each line ± SE. (C) Detection of GhCOLs expression by qRT-PCR in 35S:GhCOLs transgenic lines and co-2 under LD conditions. (D) The expression level of Arabidopsis FT was determined by qRT-PCR. Data represent the mean ± SE from three biological replicates in (C) and (D), and AtACT2 (AT3G18780) was used as internal control. ** and * indicate significant differences in comparison with co-2 mutant at P < 0.01 and P < 0.05 according to the Student’s t-test compared to mutant, respectively. (TIF) [file pone.0179038.s004.tif]
